# Biochemical and genotyping analyses of camels (*Camelus dromedaries*) trypanosomiasis in North Africa

**DOI:** 10.1038/s41598-023-34086-y

**Published:** 2023-05-03

**Authors:** Ahmed M. Darwish, Abdoallah Sharaf, Semir Bechir Suheil Gaouar, Neama I. Ali, Tamer H. Abd El-Aziz, Asmaa M. Abushady, Zoubeyda Kaouadji, Othman E. Othman, Miroslav Obornik

**Affiliations:** 1 Cell Biology Department, Biotechnology Research Institute, National Research Centre, Dokki, 12622 Giza Egypt; 2 SequAna Core Facility, Department of Biology, University of Konstanz, 78464 Konstanz, Germany; 3 Genetic Department, Faculty of Agriculture, Ain Shams University, Cairo, Egypt; 4 Applied Genetic in Agriculture, Ecology and Public Health Laboratory, SNV/STU Faculty, University of Tlemcen, Chetouane, Algeria; 5 Parasitology and Animal Diseases, Veterinary Research Institute, National Research Centre, Dokki, 12622 Giza Egypt; 6 Biotechnology School, Nile University, Sheikh Zayed, Giza Egypt; 7 Biology Centre, Institute of Parasitology, Czech Academy of Sciences, České Budějovice, Czech Republic; 8 Faculty of Science, University of South Bohemia, České Budějovice, Czech Republic

**Keywords:** Genetics, Molecular biology

## Abstract

Camels are considered an important food source in North Africa. Trypanosomiasis in camels is a life-threatening disease that causes severe economic losses in milk and meat production. Therefore, the objective of this study was to determine the trypanosome genotypes in the North African region. Trypanosome infection rates were determined by microscopic examination of blood smears and polymerase chain reaction (PCR). In addition, total antioxidant capacity (TAC), lipid peroxides (MDA), reduced glutathione (GSH), superoxide dismutase (SOD) and catalase (CAT) were determined in erythrocyte lysate. Furthermore, 18S amplicon sequencing was used to barcode and characterizes the genetic diversity of trypanosome genotypes in camel blood. In addition to *Trypanosoma*, *Babesia* and *Thelieria* were also detected in the blood samples. PCR showed that the trypanosome infection rate was higher in Algerian samples (25.7%) than in Egyptian samples (7.2%). Parameters such as MDA, GSH, SOD and CAT had significantly increased in camels infected with trypanosomes compared to uninfected control animals, while TAC level was not significantly changed. The results of relative amplicon abundance showed that the range of trypanosome infection was higher in Egypt than in Algeria. Moreover, phylogenetic analysis showed that the *Trypanosoma* sequences of Egyptian and Algerian camels are related to *Trypanosoma evansi*. Unexpectedly, diversity within *T. evansi* was higher in Egyptian camels than in Algerian camels. We present here the first molecular report providing a picture of trypanosomiasis in camels, covering wide geographical areas in Egypt and Algeria.

## Introduction

Trypanosomes are blood parasites that infect wide range of livestock^1^. Diseases caused by trypanosomes include blood dyscrasias (anemia, leukopenia, thrombocytopenia), organ damage, and inflammation^1^. Eleven different species of pathogenic trypanosomes have been studied in Africa^2^. Three species of trypanosomes are considered socioeconomically important and highly pathogenic in Africa: *Trypanosoma brucei*, *Trypanosoma evansi* and *Trypanosoma equiperdum*^3^. Recently, the complexity of the African trypanosome subspecies in nature has been explored^4^. *Trypanosoma brucei* is a group with five ecotypes, namely *T. brucei brucei, T. b. gambiense*, *T. b. Rhodesiense*, *T. b. equiperdum* and *T. b. evansi*. It is characterized by rapid evolution and can easily invade new hosts and regions due to some minor mutations, which can also be induced under laboratory conditions^4^. Parasitological and serological studies are not able to distinguish between subspecies. Therefore, various genetic and molecular methods have been used to further increase the accuracy in the diagnosing trypanosome subspecies^5^.

Oxidative stress triggered by free radicals plays an important role in the pathogenesis of trypanosomiasis in camels. Therefore, the determination of oxidants and antioxidant markers may help to determine the degree of host tissue damage caused by the infection and the health status of infected camels^6^.

Previously, direct Sanger sequencing of PCR amplicons using generic primers was considered the most common molecular method for diagnosing trypanosome infection^7^. This method is rapid and sensitive, but makes it difficult to detect multiple different co-amplified genetic variants, which means that trypanosome co-infections may go undetected, especially when one trypanosome genotype is present in lower abundance than the other^8,9^. In contrast, next-generation 18S amplicon sequencing (NGS) enables symmetric high-throughput sequencing reactions and is therefore useful for accurately determining the prevalence and genetic diversity of *Trypanosoma* subspecies in animal populations^10^. Recent advances in NGS technology enable the study of population genetics and ecology of microparasites and free-living microorganisms^11^. It also allows the parallelization of millions of sequencing reactions, enabling the investigation of species diversity and prevalence of the parasite in large populations^12^.

The aim of the present study was to genotype *Trypanosoma* spp. in Egyptian and Algerian camels (*Camelus dromedarius*) from different herds and geographical regions, and to record their morphological characteristics. Moreover, the study was conducted to evaluate the oxidative status as an indicator of oxidative damage in the erythrocytes of camels naturally infected with trypanosomes. The different trypanosome species and genotypes were identified using the 18S amplicon NGS. Phylogenetic analysis was performed to study the evolutionary relationships between the identified trypanosome genotypes and other trypanosomes.

## Methods

### Ethical approval

The experimental protocol used in the study was approved (131712012023) by the Animal Care and Use Committee of National Research Centre, Egypt in accordance with the relevant guidelines and regulations.

### Study area and samples collection

We began the study with a sampling survey of the occurrence of trypanosomiasis in native camels in Egypt and Algeria. We selected three different sampling sites in Egypt and twelve regions in Algeria where trypanosome-infected camels were expected to be present (Table S1A). We collected 1001 blood samples from Egyptian and Algerian camels: 600 from Egypt (370 samples from Marsa Matrouh, 100 samples from Aswan, 30 samples from Giza, 100 samples from Sharqia) and 401 samples from Algeria (Table S1A). Blood samples were collected in vacutainer tubes containing ethylenediaminetetraacetic acid (EDTA). The Egyptian samples were divided into two parts. The first part was used for blood smears for microscopic examination and erythrocyte lysate, whereas the second part was kept at −20 °C for DNA extraction for PCR and next generation sequencing.

### Examination of trypanosome using a thin peripheral blood smear

A thin smear was made from each sample on a microscopic slide. These slides were air dried, then fixed in methanol, and finally stained with a 10% Giemsa solution in phosphate-buffered saline (pH 7.2). All slides were examined by light microscopy using a 40× and an oil lens according to Mathison et al.^13^.

### Assessment of oxidant/antioxidant markers

The oxidant/antioxidant assessment was evaluated only in the Egyptian blood samples. The positive PCR samples for Trypanosoma (− ve Babesia and −ve Theilaria) were selected only to estimate oxidant/antioxidant parameters. The control samples were selected based on Trypanosoma-free blood smears and negative-PCR results. Also, the oxidative markers were only done in the blood lysate of camels infected with Trypanosoma. Whole blood samples were centrifuged at 3000 rpm for 10 min at 4 °C. The buffy coat and plasma were removed. The erythrocyte pellets were washed three times with an 0.9% isotonic saline solution, then centrifuged and the supernatant was removed. The washed erythrocyte pellets were lysed with nine volumes of ice-cold deionized water to prepare 10% of the erythrocyte lysate. Reduced glutathione (GSH) concentration^14^ total antioxidant capacity (TAC)^15^, hemoglobin concentration (Hb)^16^ and lipid peroxidation by-products such as malondialdehyde (MDA) content^17^ (and superoxide dismutase (SOD) activity^18^ were determined in the erythrocyte lysate using commercial kits from Biodiagnostic Co, Egypt. The activity of catalase (CAT)^19^ was measured in the erythrocyte lysate, and the decreased absorbance per min/ml after decomposition of H_2_O_2_ was considered as the CAT activity. All parameters were determined using a spectrophotometer (T80 UV/VIS PG instrument Ltd, UK).

### DNA extraction and 18S rDNA amplification

DNA was isolated from 200 μl blood sample using the QIAamp 96 DNA blood kit (Qiagen, Hilden, Germany) according to the manufacturer's instructions, and eluted in 60 μl elution buffer, and measured using Nanodrop (Thermos). Samples were screened using generic *Trypanosoma* spp. 18S rDNA PCR primers Tryp_F (5'-TGCTGTTGCTGTTAAAGGGT-3') and Tryp_R (5'-TTGTGTCTGAGTGTTCGCGG-3'). Two μl of DNA was used for each PCR reaction, and the reaction volume of 25 μl contained the following components: Taq PCR buffer (final concentration 10 mM Tris–HCl, pH 9.0, 1.5 mM MgCl2, 50 mM KCl, 0.1% Triton X-100, and 0.01% (w/v) stabilizer), 2 μM of each primer, 1 mM total dNTP’s and 1.25 U of Taq enzyme Reaction conditions were as follows: 1 cycle at 95 °C for 3 min followed by 35 cycles at 94 °C for 30 s, 55 °C for 45 s, and 72 °C for 2 min, and final extension step at 72 °C for 5 min. Ten μl of PCR product was run on a 1.5% agarose gel at 100 V. The gel was stained with ethidium bromide and visualized using a gel imaging system and a 1 kb Plus DNA ladder to calculate the relative fragment length.

### 18S amplicons preparation and sequencing

Fifteen trypanosome-positive samples represent all infected geographic regions. Camel herds in Algeria and Egypt were selected for 18S amplicon sequencing (Table S1B). Euk1391f. (5′GTACACACCGCCCGTC3′), EukBr (5′TGATCCTTCTGCAGGTTCACCTAC3′), and blocking primers (GCCCGTCGCTACTACCGATTGG/ideoxyI/TTAGTGAGGCCCT/3SpC3/) were used to amplify the V9 region under the following conditions for the primary reaction: an initial pre-amplification step at 94 °C for 3 min, followed by 35 cycles at 94 °C for 30 s, 65 °C for 15 s, 57 °C for 30 s, 72 °C for 90 s, and a final extension step at 72 °C for 10 min. Samples were amplified in triplicate, i.e., each sample was amplified in 3 replicate 25-µl PCR reactions. Triplicate PCR reactions were pooled into a single volume (75 µl) for each sample. Individual reactions were performed in 25 μl volumes consisting of 1 μl DNA, 10 μl PCR master mix (2x), 9 μl PCR water, 0.5 μl forward primer (10 μM), 0.5 reverse primer (10 μM), and 4 μl blocking primer (10 μM) (Thermo Fisher Scientific Inc.). A mammalian blocking primer (AGCCCGTCGCTACTACCGATTGG/ideoxyI//ideoxyI//ideoxyI//ideoxyI//ideoxyI/TTAGTGAGGCCCT/3SpC3/) is used to reduce the probability of host genomic DNA uptake. The C3 spacer (/3SpC3/) is a chemical modification that prevents extension during the PCR. Amplicons from each sample were run on an agarose gel and quantified using Quant-iT PicoGreen dsDNA Assay Kit. Finally, the amplicon pool was cleaned up using the MoBio UltraClean PCR Clean-Up Kit and its concentration was measured using Nanodrop (Thermos). The resulting amplicons were sequenced using the Illumina Novaseq platform (Novogene, Cambridge, UK) according to the manufacturer’s recommendations.

### Phylogenetic analysis

All available nucleotide reference sequences (390 sequences) of the small subunit (18S) ribosomal RNA gene of trypanosomes were retrieved from NCBI GeneBank (www.ncbi.nlm.nih.gov: 09:2022), which contains sequences of specimens from 35 different trypanosomes’ species/sub-species. The retrieved sequences were combined with the obtained trypaqnosome OTU sequences (3 OTUs) in a single sequence dataset.

Sequences were aligned using MAFFT software^20^ and ambiguously aligned regions were excluded for further analysis using trimAl software^21^ Alignments were tested using ProtTest v3^22^ to select an appropriate model for nucleotide substitution. Two maximum likelihood phylogenetic trees (ML) were constructed using RAxML-NG^23^ and IQ-TREE 2 software^24^. ML analyses were performed with 1000 bootstrap replicates. Supporting values from both programs are shown on the ML tree. The phylogenetic tree was rooted with Dicoba (*Leptomonas pyrrhocoris* and *Leishmania* sp.) sequences.

### Statistical analysis

Results were expressed as means ± SE for both infected and control groups. Data were statistically analyzed with independent t-tests using SPSS computer software. Differences were considered significant at the level (*P* < 0.05) and highly significant at the level (*P* < 0.001)^25^.

### Informed consent

The authors declare that they consent to participate to this study.

## Results

### Microscopic examination

**Figure 1:**
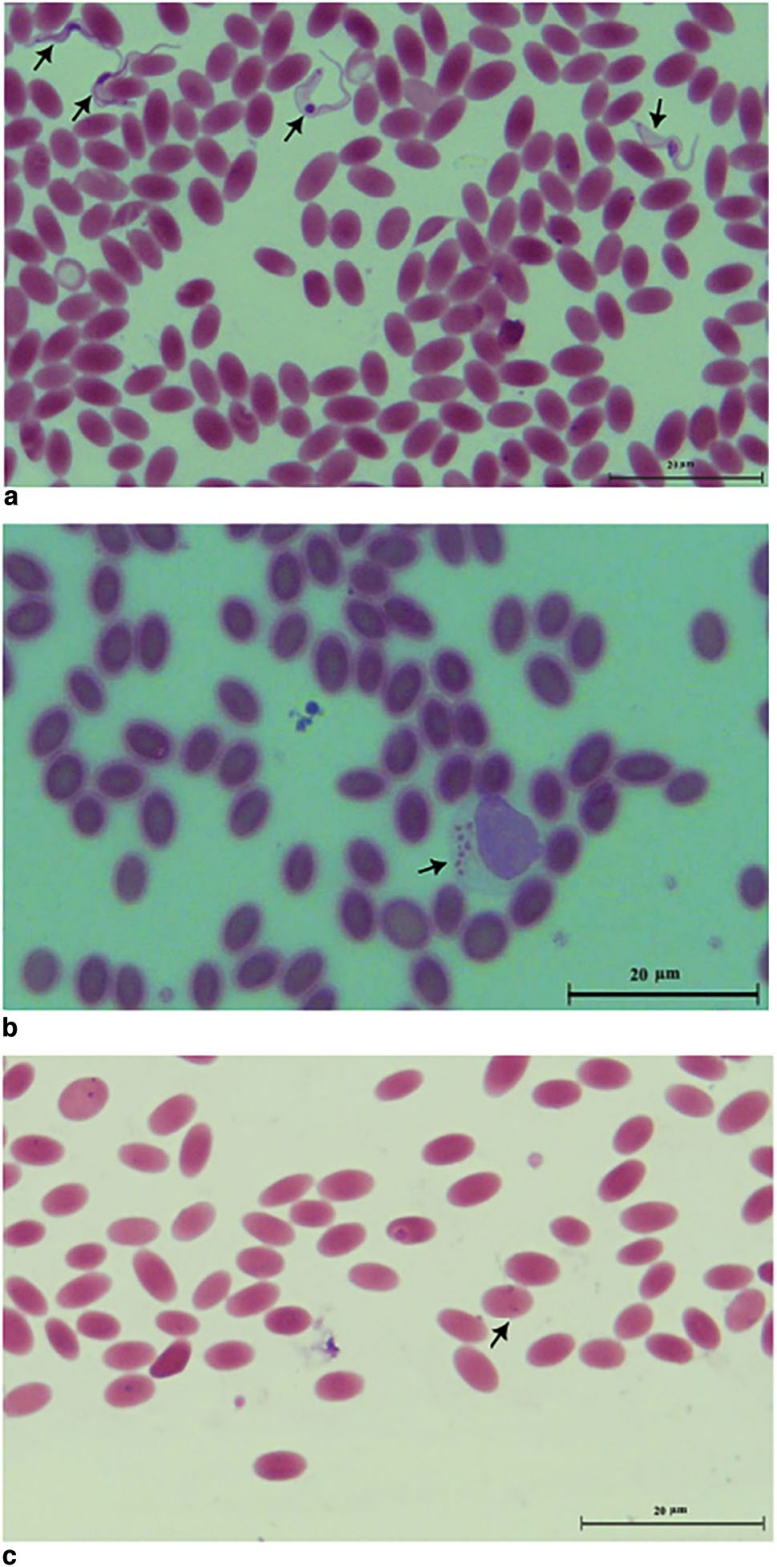
Microscopically observation of the blood films, stained with quick stainthe observation shows the presence (**a**) *Trypanosoma* spp., (**b)** Schizonts of *Theileria* spp., and (**c**) *Babesia* spp. inside lymphocyte some blood film.

Microscopic examination of the blood smear showed the presence of trypanosomes in four sample (3/370 samples collected from Mersa Matrouh governorate were positive) and (1/30 samples collected from Giza governorate were positive). The level of parasitemia in these parasitologically positive samples was relatively high, ranging from 1 to 5 trypanosomes/HPF. In addition, apicomplexan parasites *Babesia* and *Theileria* were observed in four and ten samples, respectively. The morphological characteristics of *Trypanosoma* spp*.* are slender with either tapering or blunt ends, with long free flagella and a well-developed undulating membrane (Fig. 1a). *Theileria* sp*.* on the other hand is characterized by schizonts within lymphocytes (Fig. 1b). Finally, *Babesia* sp*.* is characterized by a large trophozoite stage typically divided into a pair of merozoites (Fig. 1c).

### Biochemical analysis

We determined three biochemical indicators in the blood of the camels studied. Although the infected camel frequency in Egypt was low, at 7.2% by PCR detection (Table 1), the oxidant and antioxidant markers had significantly increased compared with infection-free camels (the control) (Table 2). Serum contents of MDA (3.46 ± 0.164) and GSH (0.30 ± 0.005) had recorded a high significant (*P* < 0.001) increase in infected camels compared with infection-free camels (1.42 ± 0.025 and 0.11 ± 0.003 respectively). Serum contents of TAC were not significantly different between infected and healthy camels (0.46 ± 0.009 vs. 0.48 ± 0.012). While the serum content of SOD (4.24 ± 0.113) and CAT (0.18 ± 0.007) had recorded a significant increase in infected camels compared with healthy camels (3.31 ± 0.044 and 0.06 ± 0.003).

**Table 1:** Trypanosome Infection percent in the Egyptian and Algerian regions based on the PCR diagnosis.

Egypt	Samples no	Positive number	Percent
Location			
Mersa Matrouh	370	40	10.8
Aswan	100	2	2
Giza	30	1	3.3
Sharqia	100	0	0
Total	600	43	7.2
Algeria			
Bechar	23	13	56.5
Masila	2	0	0
Namma	4	1	25
Bayedh	16	10	62.5
Tindouf	33	17	51.5
Adrar	30	19	63.3
Tam	85	43	50.6
Laghuouat	26	0	0
ouargla	114	0	0
Gradaia	15	0	0
Oued	27	0	0
Biskra	26	0	0
	401	103	25.7

**Table 2:** Oxidant/antioxidant markers in erythrocytes lysate (mean ± SE) in *T. evansi* infected and healthy control animals.

Parameters	Control	Infected
TAC (mmol/l)	0.48 ± 0.012	0.46 ± 0.009
MDA (nmol/ml)	1.42 ± 0.025	3.46 ± 0.164**
GSH (µg/mg Hb)	0.11 ± 0.003	0.30 ± 0.005**
SOD (U/mg Hb)	3.31 ± 0.044	4.24 ± 0.113*
CAT (µmol H2O2 decomposes/min/ml)	0.06 ± 0.003	0.18 ± 0.007*

*Significant (*P* < 0.05).

**Highly significant (*P* < 0.001).

### Diagnostic PCR

**Figure 2:**
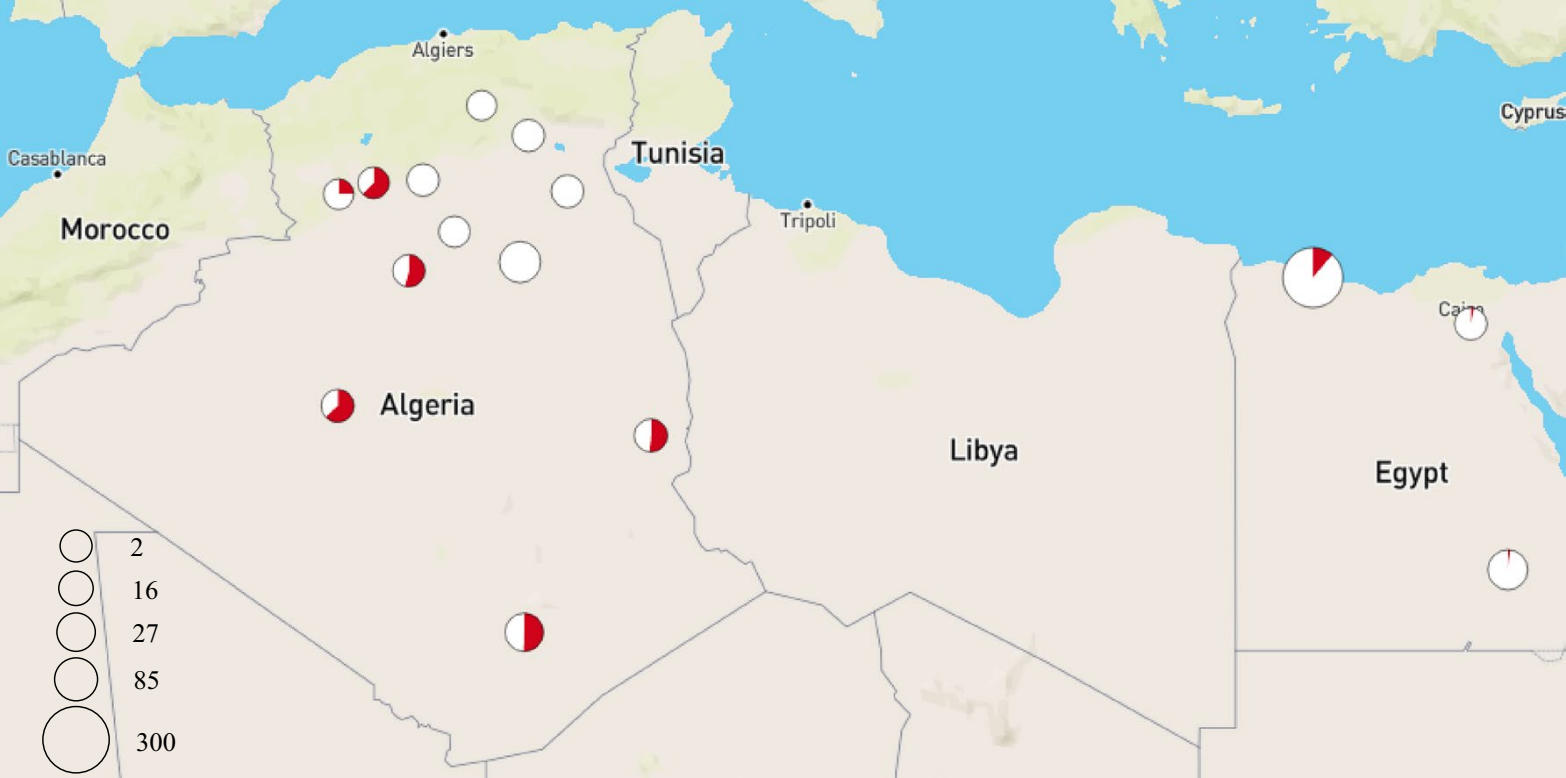
A geographic plot was generated using microreact tool (https://microreact.org), indicating the distribution of camel blood sampling sites from 3 different geographic localities in Egypt and other 12 different geographic locations in Algeria. The plot shows also the number of collected samples per site as relative size and the infection rate in each site is indicated.

Polymerase chain reaction results showed that 43 (10%) of the 426 samples collected from the Egyptian regions were trypanosome positive (Table 1 and Fig. 2). Moreover, the highest infection rate was recorded in Mersa Matrouh (10.8%), while the lowest was 2% in Aswan (Table 1 and Fig. 2). On the other hand, 103 of the 402 samples (25.6%) collected from the Egyptian regions were trypanosome positive. The highest infection rate with trypanosomes within the Algerian regions was 63.3% in Adrar and the lowest was 25% in Namma. Interestingly, no trypanosome infection was detected in the samples from Masila, Laghuouat, Ouargla, Gradaia, ELOued and Biskra collection sites (Table 1 and Fig. 2).Biochemical analysis.

### 18S amplicons data analysis

**Figure 3:**
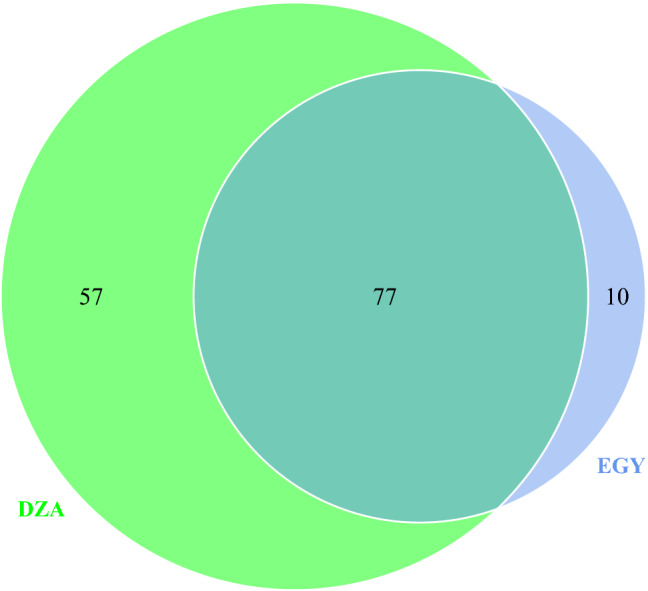
Venn diagram shows the overlaps and differences of the identified OTUs in Egyptian and Algerian selected samples for 18S amplicon sequencing.

A total of 30,000–500,000 reads were obtained from each sample. Reads were prepared, filtered, and OUTs were generated using USEARCH software^26^). In total, we identified 271 OTUs and we assigned their taxonomy using BLAST search against SILVA 18S small subunit database version 132^27^. Finally, the relative abundance of amplicons of the identified OTUs in each sample was calculated. Out the 271 OTUs generated only 3 OTUs (OTU_9, OTU_178, and OTU_256) could be taxonomically assigned to trypanosomes. In addition, we were able to assign all three OTUs to *Trypanosoma evansi*. The diversity of OTUs in the samples showed that 77 OTUs were shared between the Egyptian (Egy) and Algerian (DZA) infected samples, including 2 trypanosome OTUs (OTU_9 and OTU_178) (Fig. 3). Interestingly, 10 OTUs were specific to the Egyptian infected samples, including the trypanosome OTU (OTU_256), while 57 OTUs were specific to the Algerian samples and did not contain any trypanosome OTUs (Fig. 3).

**Figure 4:**
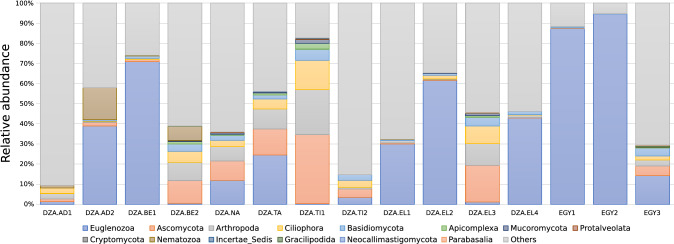
Relative Abundance of the OTUs in Egyptian and Algerian selected samples for 18S amplicon sequencing, shows the diversity of different parasites in Egyptian and Algerian camels including trypanosomes.

The results of relative amplicon abundance showed that the range of trypanosome infection was higher in Egypt (20–90%) than in Algeria (1–70%) (Fig. 4). The Egyptian samples (EGY1 and EGY2) had the highest relative abundance of trypanosomes within all samples. Samples EGY1 and EGY2 were from Marsa Matrouh and Barqash regions and from Sudani and Maghrabi camel herds, respectively, while the Egyptian samples from the Aswan region (EGY3) had a low relative abundance of trypanosomes (~ 15%) (Table S1B and Fig. 4). Moreover, the samples with the highest relative abundance of trypanosomes in the Algerian samples (DZA.AD2, DZA.BE1, DZA.EL1, and DZA.EL2) were from different camel herds in the Adrar, Bechar, and El-Bayedh regions, while the samples with the lowest relative abundance of trypanosomes were from the Adrar, Bechar, Tindouf, and El-Bayedh regions (Table S1B and Fig. 4). Interestingly, the samples with the highest and lowest relative abundance of trypanosomes were from the same geographic regions (Adrar, Bechar, and El-Bayedh) but from different camel herds (Table S1B and Fig. 4).

In addition, sequence analysis of the 18S amplicons of selected trypanosome positive samples showed that Egyptian camels were infected with a low relative abundance of other parasites, while Algerian camels were infected with high relative abundance of other parasites such as *Ascomycota*, *Arthropoda*, *Ciliophora*, *Basidiomycota*, *Apicomplexa*, *Mucoromycota*, *Protalveolata*, *Cryptomycota*, *Nematozoa*, *Gracilipodida*, *Neocallimastigomycota*, and *Parabasali* beside *Euglenozoa* (Fig. 4).

### Phylogenetic analysis

**Figure 5:**
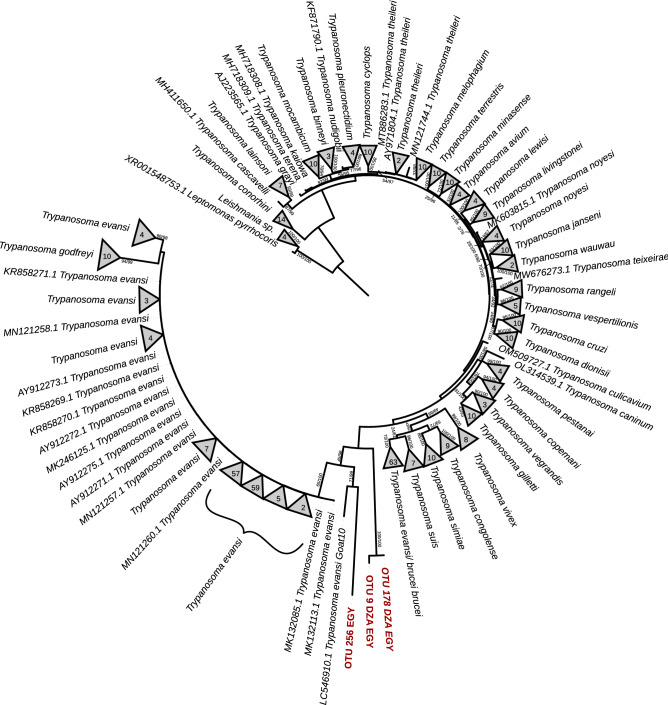
A maximum likelihood (ML) phylogenetic tree showing the evolutionary relationships of between the identified trypanosome OTUs sequences (in red), and 390 the Trypanosomes’ small subunit (18S) ribosomal RNA gene reference sequences retrieved from NCBI database (09/2022). The ML branch support values are given in % (IQ-TREE/RAxML-NG), and the number of leaves of the collapsed clade is mentioned. In addition, *Babesia vogeli*’s sequences (5 sequences) were retrieved as an out-group.

In our phylogenetic tree, all trypanosome sequences were clustered in paraphilic clades without admixture (Fig. 5). The phylogenetic tree showed that all the trypanosome OTUs identified in this study grouped in a clade in the root of the *Trypanosoma evansi* (Fig. 5). The Egyptian-specific OTUs form a sister group with *Trypanosoma evansi*, while the common OTUs between Egyptian and Algerian samples were clustered together (Fig. 5).

**Figure 6:**
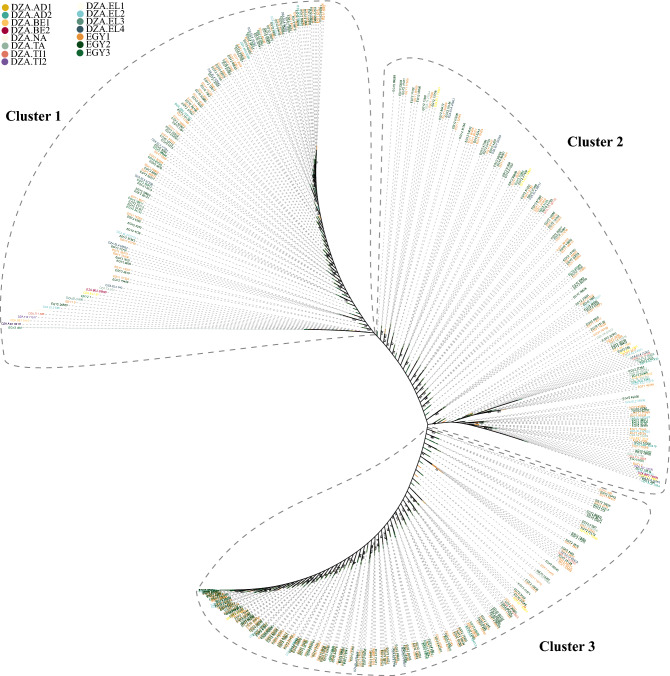
A maximum likelihood (ML) eukaryotic phylogenetic tree of the identified Trypanosomes-Amplicon Sequence Variants (ASV) in each selected trypanosome-positive sample. The ML branch support values are given in % (IQ-TREE/RAxML-NG).

To better understand the relationships between the different *Trypanosoma evansi* genotypes in the sequenced samples, the trypanosome Amplicon Sequence Variants (ASV) were identified in each sample (Table S1C). Subsequently, the identified trypanosome ASV sequences were extracted and a phylogenetic tree was constructed in the same manner as described above. The unrooted trypanosome ASV phylogenetic tree grouped all ASV sequences into three clusters but failed to cluster any of the ASV samples (Fig. 6). All ASV samples were assigned to the three clusters except for the ASV sequences of some Algerian samples (DZA.AD1, DZA.BE2, DZA.TI1, and DZA.EL4) (Fig. 6).

## Discussion

*T. evansi* is the most widespread parasite in camel blood, causing significant morbidity and mortality in camels and ranking first in among camel diseases; in particular, it was currently associated with secondary bacterial and parasitic infections^28^. In this study, suspected trypanosomiasis was investigated during blood collection. Although the study lasted two years and was conducted in different locations in Egypt and Algeria, the occurrence of trypanosomiasis seems to be similar and no significant difference was found by PCR diagnosis. However, the PCR method was recommended for trypanosomiasis diagnosis^29^. This could be due to the lack of medical care and veterinary services^30^. A similar explanation was reported from Sudan^31^. Detection of parasites in blood is difficult because parasitemia is recurrent and may be below the limit of detection of microscopic examination, and therefore positive cases are likely to be missed. In the only four microscopically positive samples, parasitemia was relatively high and ranged from 1 to 5 trypanosomes / HPF, indicating the acute phase of infection. The present study showed that the PCR method had a higher sensitivity in the diagnosing trypanosomiasis than microscopic examination of blood smears^31^ which have unacceptably low sensitivity especially in the chronic phase. The infection rate of *Trypanosoma* spp*.* infection was 10% in Egypt and 25.6% in Algeria (Table S1A and Fig. 2). The present data showed a significant increase in the level of MDA in erythrocytes as a lipid peroxidation biomarker in the camels infected by trypanosomes compared to the healthy camels. This may be due to the increased levels of free radicals as a result of trypanosome infection (Table 2). The present result is consistent with *T. evansi* infected camels^6^, horses^32,33^, buffaloes^34^ and rats^35^. Due to its function as an O_2_ and CO_2_ transporter, erythrocytes are constantly exposed to free radicals. Therefore, they have strong antioxidant defenses that convert free radicals into intermediates that are much less reactive^36^. GSH, SOD and CAT are considered to be strong antioxidant defense systems in erythrocytes. The dismutation of superoxide anion (O_2_^-^) and hydrogen peroxide (H_2_O_2_) was catalyzed by SOD, while GSH and CAT catalyze the degradation of H_2_O_2_ into H_2_O and O_2_^37^. Our results showed a significant increase in GSH level, and the activities of SOD and CAT were observed in infected camels and not in healthy camels. Increased levels of SOD and CAT have been reported previously^34,38^. This may be due to stimulation of the antioxidant system to remove free radicals generated by trypanosome infection. In previous studies camels^6^, horses^33^ and rats^35^ infected with *T. evansi* showed a significant decrease in GSH, SOD and CAT concentrations, which was explained by the depletion of their stores due to oxidative stress.

On the other hand, a high parasite diversity was observed with 271 OTUs in more than 15 parasite families covering the most important parasites identified in Egypt and Algeria in fauna: *Euglenozoa*, *Ascomycota*, *Arthropoda*, *Ciliophora*, *Basidiomycota*, *Apicomplexa*, *Mucoromycota*, *Protalveolata*, *Cryptomycota*, *Nematozoa*, *Gracilipodida*, *Neocallimastigomycota*, and *Parabasalia*. The 271 different OTUs identified are thought to represent the intraspecific diversity of Egyptian and Algerian parasites. This diversity should be taken with caution, as the Illumina platform has a low error rate^39^, and errors are common in PCR. However, the error was calculated using UNOISE2 to filter and group the sequences into OTUs to generate more biologically relevant sequence data^26^. Indeed, the present 18S amplicon sequencing results showed an unexpectedly high diversity within *T. evansi* isolated from Egyptian camels more than from Algerian camels (Table S1C and Fig. 6). The large number of genetic variants could reflect the long period of isolation in Egypt and Algeria or indicate a wide range of vectors^40,41^. This increase in diversity of *T. evansi* could also indicate genetic exchange that occurs in several other trypanosomes such as *T. brucei*^42^ and *T. cruzi*^43^, leading to the formation of hybrid species that increase genetic diversity.

Many factors could explain the observed differences between trypanosomes in Egyptian camels and Algerian camels, e.g., environmental and physiological differences, stress, and immune function may also contribute to more diverse parasite infections^44^. Although there is no evidence that immune function is compromised in Egyptian camel populations, a collaborative study with woylies (*Bettongia penicillata*) using fecal cortisol metabolite (FCM) concentrations as an indicator of stress found higher FCM concentrations in woylies (both transported and resident animals) after translocation^44^. Finally, epidemiological factors such as population size and status among species could also lead to differences in their exposure to trypanosome vectors or susceptibility to infection^41^. Although all trypanosomes can be detected early in the blood due to their ease of decomposition^45^, it is not possible to distinguish the species due to their close similarity in appearance, so they have been ranked as the currently accepted subspecies (*T. brucei*, *T. brucei gambiense*, and *T. brucei rhodesiense*)^46^. *Trypanosoma brucei evansi* and *T. b. equiperdum* were considered separate species for more than a century^5^. Since there are no obvious differences from *T. b. brucei* in their nuclear genome^47^, the complete loss or deletions of mitochondrial DNA makes *T. b. evansi* and *T. b. equiperdum* as petite mutants 89^47,48^. Although *T. b. rhodensiense* differs by the serum resistance-associated (SRA) gene, this gene is being shared between *T. b. rhodesiense* and *T. b. brucei* in East Africa^49,50^. *Trypanosoma brucei gambiense*-specific genes such as glycoprotein (TgsGP)^51^, have been identified in some strains of *T. b. rhodesiense* and *T. b. brucei*. In addition, the *T. b. evansi*-specific variant surface glycoproteins (VSGs)^52,53^ have been reportedly identified recently in several strains of *T. b. brucei* and *T. b. gambiense*^54^. Therefore, ambiguous distinctions between *T. b. brucei*, *T. b. gambiense*, and *T. b. rhodensiense* are tenuous at best and depend largely on the evolution of VSGs^4^. Our study has shown that next generation sequencing (NGS) technologies are very useful in determining diversity in a community, as they allow identification of multiple parasites and more accurate estimation of their prevalence, as well as detection of different genotypes for trypanosomes.

## Conclusion

Polymerase chain reaction was more sensitive than microscopic observations in diagnosing trypanosomes. The prevalence of trypanosome infection was found to be higher in Algeria than in Egypt. In addition, there was a highly significant increase in MDA, GSH, SOD and CAT levels in the blood of trypanosome-infected camels compared to the uninfected control, while the TAC level was not significantly changed. The sequence analysis of the 18S amplicons of the positive samples showed that the range of trypanosome infection was higher in Egypt than in Algeria with a low relative abundance of other parasites. Although the results of phylogenetic analysis showed that some *Trypanosoma* sequence of Egyptian camel samples formed a separate group from the group that combined the rest of the Egyptian camel samples with the Algerian camel, the two groups were related to *T. evansai*. Finally, all trypanosome ASV sequences were clustered into three clades with no evidence of ASV or sample clade specificity.

## Supplementary Information

Supplementary Information 1.

Supplementary Information 2.

## Data Availability

The fifteen datasets of 18S amplicon sequencing are available at the NCBI Short Read Archive (SRA) with the GenBank Accession No.: PRJEB55278.
