# Supplementary material for: Biochemical and genotyping analyses of camels (Camelus dromedaries) trypanosomiasis in North Africa
Source: Sci Rep. 2023 May 3;13:7176. doi: 10.1038/s41598-023-34086-y (PMC10156784; doi:10.1038/s41598-023-34086-y)
Supplement: Supplementary file 2 — Supplementary Information 2. [file 41598_2023_34086_MOESM2_ESM.docx]

**Supplementary Materials legend**

**Table S1. A)** A list of the studied camel blood samples shows the sampling sites, the Global Positioning System (GPS) points, and sample codes. Moreover, the table shows the results of PCR diagnosis and the animal's sex and population/herd when it is known. **B)** A list of the selected trypanosome-positive samples for 18S amplicon sequencing shows the same metadata as the previous table. **C)** Number of the typanosomes-Amplicon Sequence Variants (ASV) identified in each selected trypanosome-positive sample.
